# Genome-Wide Screen for Modifiers of Ataxin-3 Neurodegeneration in *Drosophila*

**DOI:** 10.1371/journal.pgen.0030177

**Published:** 2007-10-19

**Authors:** Julide Bilen, Nancy M Bonini

**Affiliations:** 1 Department of Biology, University of Pennsylvania, Philadelphia, Pennsylvania, United States of America; 2 Howard Hughes Medical Institute, University of Pennsylvania, Philadelphia, Pennsylvania, United States of America; University of Minnesota, United States of America

## Abstract

Spinocerebellar ataxia type-3 (SCA3) is among the most common dominantly inherited ataxias, and is one of nine devastating human neurodegenerative diseases caused by the expansion of a CAG repeat encoding glutamine within the gene. The polyglutamine domain confers toxicity on the protein Ataxin-3 leading to neuronal dysfunction and loss. Although modifiers of polyglutamine toxicity have been identified, little is known concerning how the modifiers function mechanistically to affect toxicity. To reveal insight into spinocerebellar ataxia type-3, we performed a genetic screen in *Drosophila* with pathogenic Ataxin-3-induced neurodegeneration and identified 25 modifiers defining 18 genes. Despite a variety of predicted molecular activities, biological analysis indicated that the modifiers affected protein misfolding. Detailed mechanistic studies revealed that some modifiers affected protein accumulation in a manner dependent on the proteasome, whereas others affected autophagy. Select modifiers of Ataxin-3 also affected tau, revealing common pathways between degeneration due to distinct human neurotoxic proteins. These findings provide new insight into molecular pathways of polyQ toxicity, defining novel targets for promoting neuronal survival in human neurodegenerative disease.

## Author Summary

Spinocerebellar ataxia type-3 is the most common dominantly inherited movement disorder and is caused by a CAG repeat expansion within the gene *ATXN3*, encoding the Ataxin-3 protein. This leads to a protein with an expanded polyglutamine domain, which confers a dominant toxicity on the protein, leading to late onset, progressive neural degeneration in the brain. Although some modifiers of Ataxin-3 toxicity have been defined, little was known about their molecular mechanisms of action. The fruit fly *Drosophila* recapitulates fundamental aspects of the human disease. Here, we performed a genome-wide screen for new modifiers of Ataxin-3 toxicity using the fly and defined 25 modifiers in 18 genes. The majority of the genes belong to chaperone and ubiquitin proteasome pathways, which modulate protein folding and degradation, but the remaining modifiers have a broad range of predicted molecular functions. Assays in vivo revealed that the biological activity of all modifiers converge on aiding in situations of protein misfolding, despite distinct predicted molecular functions. Select modifiers of Ataxin-3 toxicity also modulated tau toxicity associated with Alzheimer disease. These findings underscore the importance of protein homeostasis pathways to disease and provide the foundation for new therapeutic insight.

## Introduction

Spinocerebellar ataxia type 3 (SCA3) is the most common dominantly inherited ataxia worldwide and is caused by a CAG repeat expansion encoding glutamine within the *ATXN3* gene [1,2]. The expanded polyglutamine (polyQ) is thought to confer toxicity on the protein Ataxin-3, leading to neural dysfunction and loss [3]. At least nine human diseases, including additional spinocerebellar ataxias and Huntington's disease, share this mechanism. Studies on such pathogenic proteins reveal that the long polyQ domain alters protein conformation, causing an enriched beta sheet structure [4]. Mutant polyQ protein also dynamically associates with chaperones and colocalizes with proteasome subunits, indicating that the protein is misfolded or abnormally folded [5,6]. Such accumulation of misfolded protein is a common pathology of human degenerative disorders, including Alzheimer, Parkinson, and prion diseases [7–9], indicating that these diseases may share molecular and cellular mechanisms.

Models for human neurodegenerative diseases in simple systems have provided valuable tools to dissect molecular mechanisms of disease pathology [10–13]. Directed expression of pathogenic human Ataxin-3 in *Drosophila* recapitulates key features of disease, with late-onset neuronal dysfunction and degeneration accompanied by ubiquitinated inclusions [14,15]. Neurotoxicity is more severe with increasing length of the polyQ repeat, similar to the human disease where longer repeats are associated with more severe and earlier onset disease [16,17]. In the fly, increased levels of expression of the disease protein leads to more severe degeneration and earlier onset protein accumulation, suggesting that abnormal accumulation of the mutant protein is central to disease and degeneration.

A number of modifiers of select polyQ disease proteins have been identified using animal models, including chaperones, transcriptional coregulators, and microRNAs [18–22]. Although these approaches have revealed genes that modulate polyQ toxicity, little is known regarding how the modifiers act biologically to modulate polyQ degeneration. Among polyQ proteins, Ataxin-3 is unique because it has been implicated in ubiquitin pathways, and its normal activity may impinge on protein degradation pathways [23–26]. Truncation of Ataxin-3 to remove the ubiquitin protease domain, or mutation of the ubiquitin protease activity, dramatically enhances toxicity [15], indicating that the normal activity of Ataxin-3 may be critical in Ataxin-3-induced degeneration. To reveal insight into pathways that modulate Ataxin-3 neurodegeneration, we performed a genetic modifier screen in *Drosophila*. These studies revealed a range of modifiers that, despite some broadly diverse predicted molecular functions, converge on protein misfolding with a subset mitigating toxicity through proteasome and/or autophagy pathways. These findings underscore the critical role of protein quality control in SCA3 pathogenesis and provide potential new targets toward therapeutics.

## Results

### A Genetic Screen Defines Modifiers of Pathogenic Ataxin-3 Toxicity

**Figure 1:**
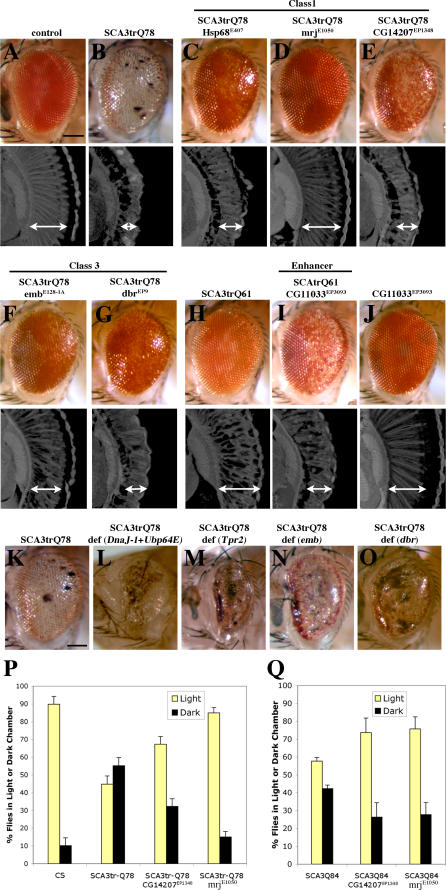
Modifiers of SCA3trQ78-Induced Neurodegeneration in *Drosophila* Eye and Internal Retinal Sections of 1-d Flies (A) Control with normal eye and retinal thickness (white arrow). Genotype *w; gmr-GAL4/+.* (B) Expression of strong SCA3trQ78 causes external and internal degeneration, with pigment loss and severely reduced retinal thickness. Genotype *w; gmr-GAL4 UAS-SCA3trQ78/+*. (C–E) Class 1 suppressors *Hsp68^E407^*, *mrj^E1050^*, and *CG14207^EP1348^* rescued external eye pigmentation and restored the internal eye towards normal. Genotypes *w; gmr-GAL4 UAS-SCA3trQ78* in *trans* to alleles indicated. (F and G) Class 3 suppressors *emb^E128-1A^* and *dbr^EP9^* have strong and weak rescue. Genotypes *w; gmr-GAL4 UAS-SCA3trQ78* in *trans* to alleles indicated. (H–J) Co-expression of *CG11033^EP3093^* with *SCA3trQ61* severely enhances pigment loss and internal retinal degeneration; expression of *CG11033^EP3093^* alone has no effect. Genotypes (H) *w; gmr-GAL4 UAS-SCA3trQ61/+*, (I) *w; gmr-GAL4 UAS-SCA3trQ61* in *trans* to *CG11033^EP3093^*, and (J) *w; gmr-GAL4* in *trans* to *CG11033^EP3093^*. (K–O) Deletions for regions that uncover select modifier genes enhance Ataxin-3 degeneration, suggesting dose sensitivity of these modifiers. (K) Pathogenic Ataxin-3 causes neuronal degeneration. (L) Ataxin-3 in *trans* to *Df(3L)CH20*, which uncovers both *DnaJ-1* and *Ubp64E*, is lethal and causes severe degeneration (pupal eye shown). (M–O) Ataxin-3 in *trans* to deficiencies uncovering *Tpr2* (Df(2L)r10), *emb* (Df(2L)TE29A), or *dbr* (Df(2L)PM44). Genotypes (K) *w; gmr-GAL4 UAS-SCA3trQ78/+*, (L) *w; gmr-GAL4 UAS-SCA3trQ78 /+; Df(3L)CH10/+*, (M) *w;gmr-GAL4 UAS-SCA3trQ78/ Df(2L)r10*, (N) *w;gmr-GAL4 UAS-SCA3trQ78/ Df(2L)TE29A*, and (O) *w;gmr-GAL4 UAS-SCA3trQ78/ Df(2L)PM44*. (P and Q) Modifiers functionally restore vision. (P) Flies with normal vision chose light when given a choice between light and dark chambers; flies expressing SCA3tr-Q78 are blind, choosing light and dark in equal numbers (1-d flies). Co-expression of *mrj^E1050^* or *CG14207^EP1348^* with SCA3trQ78 restored vision toward normal. Genotypes: Canton-S, *w; gmr-GAL4 UAS-SCA3trQ78* in *trans* to *+, mrj^E1050^* or *CG14207^EP1348^.* (Q) Modifiers mitigate vision loss induced by full-length pathogenic Ataxin-3. Flies expressing SCA3-Q84 have strikingly compromised vision (7-d flies). Coexpression of *CG14207^EP1348^* or *mrj^E1050^* suppressed visual defects and restored phototaxis. Mean ± standard error of the mean of three experiments. Genotypes *w; gmr-GAL4 UAS-SCA3Q84* in *trans* to *+, mrj^E1050^* or *CG14207^EP1348^.* Bar in (A), 100 μm for (A–J); bar in (K), 100 μm for (K–O).

To define modifiers that may reveal new insight into mechanisms of human SCA3 disease, we performed an overexpression screen for modifiers of Ataxin-3-induced neurodegeneration in *Drosophila*. SCA3trQ78 causes late onset progressive degeneration characterized by loss of pigmentation and collapse of the eye (Figure 1A and 1B) [14]. We initially screened a subset of 2,300 available *EP*-element insertion lines, each carrying a transposon engineered to direct expression of the downstream gene in the presence of the yeast GAL4 protein [27]. Because reproducibility was variable with this collection, we then performed a screen de novo, selecting for new *EP*-insertions that modified SCA3trQ78 toxicity. This approach identified 17 suppressors and one enhancer (Figures 1C–1J and S1), which affected both external and internal retinal degeneration.

Plasmid rescue was performed to identify the genes affected. BLAST searches with genomic sequence from the integration sites revealed that the lines bore insertions in the 5′ regulatory region of select genes, and all were in an orientation to direct GAL4-dependent gene expression (Text S1; Table S1). Northern and reverse transcription PCR analysis confirmed upregulation of the targeted genes; using a variety of tests we confirmed that the modifiers did not appear to affect transcription of the Ataxin-3 transgene or general GAL4-UAS transgene expression (Figure S2). Both molecular and genetic analyses confirmed that the insertions were single insertions (see Materials and Methods). Reversion analysis proved that the *EP*-elements were causal in modification. Taken together, these data indicated that the *EP* modifiers resulted in increased expression of the targeted downstream genes, which modulated SCA3trQ78 toxicity and neurodegeneration.

### Molecular Analysis Implicates Diverse Biological Pathways

Analysis of the targeted genes revealed that the majority fell into two major classes of chaperones and ubiquitin-pathway components (Table 1; Figures S1 and S3). The remaining modifiers were placed in a third category of miscellaneous functions. Class 1 (Figure 1C–1E) included an Hsp70 family member, *Hsp68^E407^*; two Hsp40 genes, *DnaJ-1^B345.2^* and *mrj^E1050^*; a small heat shock protein αB crystalline *CG14207^EP1348^*; and the cochaperone *Tpr2^EB7-1A^*. The class 2 ubiquitin-pathway components included: polyubiquitin *CR11700^EP1384^*; a ubiquitin-specific protease *Ubp64E^E213-1A^*; two ubiquitin ligases, *CG8209^B3-Sa^* and *Faf ^E659^*; and an F-box protein *CG11033^EP3093^*, the only enhancer of the group. Class 3 (Figure 1F and 1G) included genes with a variety of predicted molecular functions: the nuclear export protein Embargoed, *emb^E2-1A^* and *emb^E128-1A^*; three transcriptional regulators *Sin3A^B9-E^*, *NFAT* (*NFAT^EP1335^* and *NFAT^EP1508^*), and *debra* (*dbr^EP456^* and *dbr^EP9^*); three translational regulators, including four alleles of the *lin-41* homologue *dappled* (*dpld^JM120^*, *dpld^JM265^*, *dpld^EP546^*, and *dpld^EP2594^*), a polyA binding protein *orb2^B8-S^*, and insulin growth factor II mRNA binding protein *Imp^EP1433^*; and finally a fatty acid oxidation enzyme palmitoyl co-A oxidase *CG5009^B227.2^*. This indicated that, although chaperone and ubiquitin-pathway components are major modifier categories, a variety of functional pathways are implicated in Ataxin-3 pathogenesis.

**Table 1:**
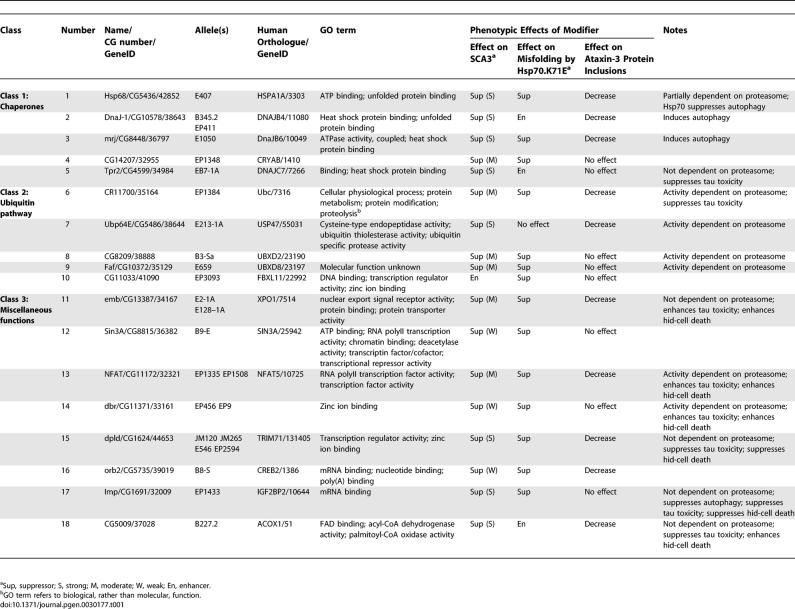
Activities of Genetic Modifiers of Ataxin-3 Neurodegeneration

The screen selected for modifiers that, upon upregulation, affected toxicity. To determine whether the activity of these genes may normally play a role in SCA3 toxicity, we examined whether reduction in the level of the modifier genes had an effect. To do this, we reduced gene expression by 50% using loss-of-function alleles where available or deficiency lines. Among these, reduction with a deficiency of *DnaJ-1* and *Ubp64E* (within the same deficiency; reduction of DnaJ-1 has previously been shown to enhance with a dominant-negative construct [16]), *Trp2*, *emb* (with an allele), and *dbr* dramatically enhanced degeneration (Figure 1K–1O; Table S2). Although the deficiencies reduce the level of a number of genes, these data suggest that the endogenous activity of these genes may normally help to protect against degeneration.

To address whether morphological rescue correlated with functional rescue, we determined the ability of select suppressors to restore function in a phototaxis assay. When flies bearing the SCA3trQ78 protein were given a choice between a light and dark chamber, they distributed randomly, indicating that they are functionally blind (Figure 1P). However, when *mrj^E1050^*, which dramatically rescued degeneration, was co-expressed, normal vision was restored. A milder suppressor that anatomically restored less retinal tissue, *CG14207^EP1348^*, restored vision partially. Thus, anatomical rescue correlated with functional rescue. These and other studies indicated that the modifiers not only modulated toxicity of the external eye, but also that of the neuronal cells. To confirm this in another situation, we tested select modifiers for the ability to mitigate polyQ toxicity when directed exclusively to the nervous system with elav-GAL4 (Figure S4). These studies confirmed that the modifiers mitigated neuronal toxicity of the Ataxin-3 protein.

We performed select additional experiments with *dpld*, for which we obtained many independent *EP* overexpression alleles. These detailed studies confirmed activity of the *EP* alleles of *dpld* with independent *UAS-dpld* transgenic lines. Further, suppression by *dpld* was not limited to development but also extended to the adult timeframe (Figures S2, S5, and S6).

Because the genes were isolated as modifiers of a truncated Ataxin-3 protein, we tested whether they could mitigate toxicity of full-length Ataxin-3, which is largely a neuronal toxicity [15]. Modifiers that phenotypically strongly suppressed toxicity of truncated Ataxin-3 also strongly suppressed full-length Ataxin-3 toxicity; however, a number of modifiers that were weak or moderate suppressors of the truncated protein were, in contrast, strong suppressors of full-length Ataxin-3: *CG14207^EP1348^* (αB crystalline), *CR11700^EP1384^* (polyubiquitin), *CG8209^B3-Sa^* (putative ubiquitin ligase), and *Sin3A^B9-E^* (Figure S7 and unpublished data). Consistent with strong anatomical rescue, *CG14207^EP1348^* also robustly suppressed functional vision defects of full-length Ataxin-3 (Figure 1Q). The enhancer, however, *CG11033^EP3093^* had a minimal effect on toxicity of full-length Ataxin-3 (unpublished data). This indicated that the modifiers varied in strength and selectivity depending upon whether the Ataxin-3 protein was intact or truncated. As truncation may be a feature of SCA3 disease [28–30], the effectiveness of modifiers against different forms of Ataxin-3 has implications for disease pathogenesis.

### Ataxin-3 Modifiers Affect General Protein Misfolding

**Figure 2:**
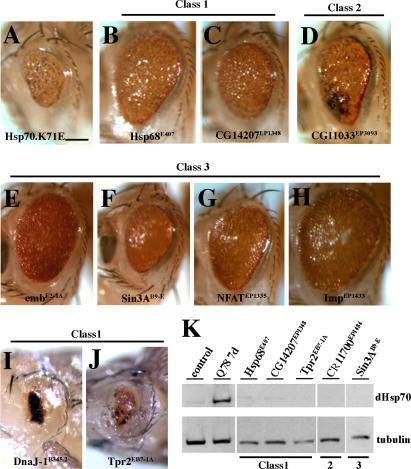
EP Modifiers Strongly Affect General Protein Misfolding Defects (A) Compromised endogenous Hsp70 via expression of a dominant negative Hsp70 transgene (*UAS-Hsp70.K71E*) leads to a degenerate eye. Genotype *w; gmr-GAL4 UAS-Hsp70.K71E/+*. (B–H) The chaperone modifiers (B) *Hsp68^E407^* and (C) *CG14207^EP1348^* partially rescue Hsp70.K71E. (D) An enhancer of Ataxin-3 toxicity (*CG11033^EP3093^*) also suppresses general misfolding, suggesting that Ataxin-3 toxicity and misfolding do not have identical molecular mechanisms. (E) *emb^E2-1A^*, (F) *Sin3A^B9-E^*, (G) *NFAT^EP1335^*, and (H) *Imp^EP1433^* also suppress the Hsp70.K71E phenotype suggesting a role of these modifiers in protein quality control. Genotypes *w; gmr-GAL4 UAS-Hsp70.K71E* in *trans* to (B) *Hsp68^E407^*, (C) *CG14207^EP1348^*, (D) *CG11033^EP3093^*, (E) *emb^E2-1A^*, (F) *Sin3A^B9-E^*, (G) *NFAT ^EP1335^*, and (H) *Imp ^EP1433^*. (I and J) Cochaperones *DnaJ-1^B345.2^* and *Tpr2^EB7-1A^* are pupal lethal upon expression with Hsp70.K71E, causing more severe misfolding phenotype. Genotype *w; gmr-GAL4 UAS-Hsp70.K71E* in *trans* to *EP* lines (I) *B345.2* and (J) *EB7-1A*. Bar in (A), 100 μm for (A–J). (K) Western blot indicates that the modifiers do not induce expression of Hsp70. Protein samples from the 1-d fly head, with indicated genotypes, *gmr-GAL4* driver. Positive control, 7-d flies expressing *UAS-SCA3trQ78*.

Previous studies have shown that the molecular chaperone Hsp70 is a potent suppressor of SCA3 toxicity [31]. Therefore, we considered that the class 1 modifiers may function similar to Hsp70 to help cells handle the misfolded disease protein, whereas those of class 2 likely have a role in ubiquitin-dependent pathways that process misfolded proteins. However, class 3 presented a range of potential activities. To address how the modifiers were functioning biologically, we tested whether the modifiers could affect a general protein misfolding phenotype: compromised chaperone activity with a dominant-negative form of Hsp70 (Hsp70.K71E). This situation results in an eye phenotype that resembles severe polyQ degeneration (Figure 2A and [32]). Thus, modifiers that affected both SCA3 and Hsp70.K71E toxicity would likely include those whose mode of action was to modulate protein misfolding.

Strikingly, we found that most of the suppressors of polyQ toxicity also mitigated the Hsp70.K71E phenotype, as well as or better than directed expression of Hsp70 itself (Figure 2; Table 1). Interestingly, *DnaJ-1^B345.2^* and *Tpr2^EB7-1A^* enhanced this phenotype; we interpreted this to indicate that these genes, which encode proteins thought to act as cochaperones of Hsp70, may compromise residual Hsp70 in the dominant-negative situation. The chaperone *mrj^E1050^*, although an Hsp40 class chaperone, acted in a manner distinct from *DnaJ-1* *^B345.2^*, as it suppressed rather than enhanced the Hsp70.K17E phenotype. Moreover, the enhancer of SCA3trQ78 toxicity, *CG11033^EP3093^*, suppressed the misfolding phenotype. Only one modifier did not affect this phenotype, the ubiquitin protease *Ubp64E^E213-1A^*; interestingly, normal Ataxin-3 also has ubiquitin protease activity that mitigates its own pathogenicity, and similarly, has no effect on Hsp70.K71E [15]. We considered that one mechanism by which the modifiers may mitigate the Hsp70.K71E phenotype is by upregulating Hsp70/Hsc70 chaperones; however, none of the modifiers appeared to act in this way (Figure 2K and unpublished data). These results indicated that the majority of modifiers of SCA3trQ78 toxicity appeared to function biologically by aiding in situations of compromised chaperone activity and/or protein misfolding.

### Reduced Accumulation of the Pathogenic Protein Reduces Toxicity

The degree of neurodegeneration induced by pathogenic polyQ protein is typically correlated with the level of accumulation of the protein in animals in vivo. We reasoned, therefore, that the modifiers may affect protein levels. We therefore examined protein accumulation by immunohistochemistry and western analysis. Although nuclear inclusions may not be causal in disease [33], later onset, smaller nuclear inclusions are typically reflective of reduced pathogenicity of the protein in vivo. Hsp70 and Hsp40 have also been shown to increase the solubility of pathogenic polyQ protein by western blots, concomitant with reducing toxicity [16]. We therefore examined protein accumulation using *rh1-GAL4* or the full-length Ataxin-3 protein—both situations that allow sensitive analysis of protein accumulation [15,22]. In these studies, we limited analysis to the strong and moderate modifiers.

**Figure 3:**
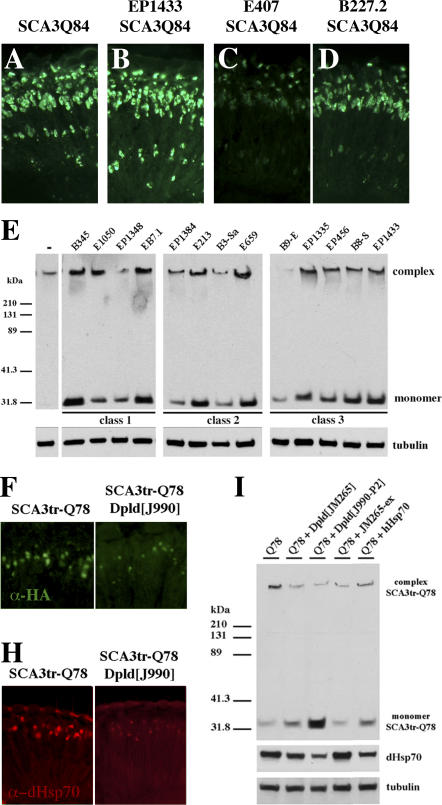
Modifiers Affect Accumulation or Solubility of Ataxin-3 Protein (A–D) Horizontal cryosections of 1-d flies indicate two modes of activity of suppressors on disease protein accumulations: reduction or no effect. (A) Control flies showing NI of full-length SCA3Q84 protein. Genotype *w; gmr-GAL4 UAS-SCA3Q84 /+*. (B) *Imp^EP1433^* has no effect on NI. Genotype *Imp^EP1433^; gmr-GAL4 UAS-SCA3Q84/+.* (C) *Hsp68^E407^* and (D) *CG5009^B227.2^* decrease NI. Genotypes (C) *w; gmr-GAL4 UAS-SCA3Q84/+; E407/+* and (D) *w; gmr-gal4 UAS-SCA3Q84 /B227.2*. (E) Western blot shows that all suppressors increase the solubility of the disease protein. Normally, no monomer is seen in the line expressing strong SCA3trQ78 (lane 1). Upon co-expression of suppressors, monomer level of disease protein increases, indicative of increased solubility. Genotype *w; gmr-GAL4 UAS-SCA3Q84* in *trans* to indicated suppressors. (F and H) Horizontal retinal sections of 7-d flies. (F) NI in flies expressing SCA3trQ78 are strongly reduced by Dpld. (H) Endogenous Hsp70 stress response is also strongly reduced by Dpld. Genotypes (F–H left panel) *w; UAS-SCA3trQ78/+; rh1-GAL4* /+ and (F–H right panel) *w; UAS-SCA3trQ78/+; rh1-GAL4* / *dpld ^J990-P2^*. (I) Western blots of 7-d flies showing that reduced stress response, as determined by levels of endogenous Hsp70, correlates with increased solubility of disease protein. Dpld increases disease protein monomer level and reduces stress-induced Hsp70. Genotype *w; UAS-SCA3trQ78; rh1-GAL4* in *trans* to +, *dpld^J990-P2^*, *dpld^JM265^, dpld^JM265ex^*, and *hHsp70*.

Immunohistochemical analysis revealed that select modifiers had striking effects to reduce NIs. These included the class I chaperones *Hsp68^E407^*, *DnaJ-1^B345.2^*, *mrj^E1050^*, but did not include αB-crystalline *CG14207^EP1348^* or *Tpr2^EB7-1A^* (Figure 3; Table 1). Of the class 2 ubiquitin-pathway components, polyubiquitin *CR11700^EP1384^* and the ubiquitin protease *Ubp64E^E213-1A^* reduced NIs, but the other strong modifiers of this class did not. Of class 3 modifiers tested, all reduced NI except *Imp^EP1433^* (Figure 3B and 3F). We then analyzed solubility of the pathogenic protein by immunoblot. This approach revealed that, although the modifiers had no effect on protein levels at early time points prior to inclusion formation, all suppressors increased the level of monomeric protein over time, thus all increased the solubility properties of the pathogenic protein (Figure 3E). The effect was specific, as co-expression of a control protein (green fluorescent protein [GFP]) had no effect (unpublished data). Similarly, the enhancer reduced monomer levels (unpublished data). These findings indicate that the modifiers either affected pathogenic protein accumulation or altered the solubility properties of the toxic protein, concomitant with altering protein pathogenicity.

### Select Modifiers Are Sensitive to In Vivo Inhibition of the Proteasome

**Figure 4:**
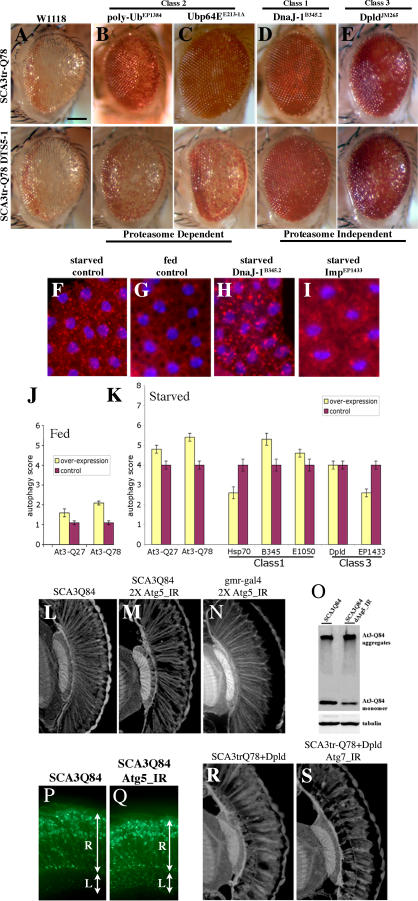
Degradation Pathways of Ataxin-3-Induced Neurodegeneration (A–E) External eyes of 1-d flies showing suppression of SCAtrQ78 toxicity by modifiers only (upper panel), and suppression by modifiers in limited proteasome background (lower panel) by expression of a dominant negative form of a 20S proteasome subunit [35]. (A) Limiting proteasome activity alone does not enhance Ataxin-3 degeneration. Genotype (upper panel) *w; gmr-GAL4 UAS-SCA3trQ78 /+* and (lower panel) *w; gmr-GAL4 UAS-SCA3trQ78/+ ; UAS-DTS5/+.* (B and C) Polyubiquitin *CR11700^EP1384^* (B) and *Ubp64E^E213-1A^* (C) cannot suppress when proteasome activity is limited. Genotypes (B) *EP1384; gmr-GAL4 UAS-SCA3trQ78 /+; UAS-DTS5/+* and (C) *w; gmr-GAL4 UAS-SCA3trQ78/+; UAS-DTS5/E213-1A.* (D and E) *DnaJ-1^B345.2^* and *dpld^JM265^* suppression remain largely unaffected when proteasome activity is limited. Genotypes (D) *w; gmr-GAL4 UAS-SCA3trQ78/+; UAS-DTS5/ B345.2* and (E) *w; gmr-GAL4 UAS-SCA3trQ78/JM265; UAS-DTS5/+.* (F–I) Fat body tissues from larvae under starved or fed conditions, stained by Lysotraker Red (red) to highlight lysosomes and Hoescht (blue) for nuclei. Starvation strongly induces lysosomes (F), whereas fed animals normally have limited lysosomes (G). Ubiquitous expression of *DnaJ-1* upregulates the induction of lysosomes in the fat bodies in starvation conditions (H), suggesting that *DnaJ-1* may reduce NI in a disease situation by facilitating autophagy. *Imp* suppresses starvation-induced autophagy (I) although has no effect on NI, raising the possibility that it may modulate autophagic cell death. Genotypes (F–G) *w*, (H) *w; da-GAL4/B345.2*, and (I) *EP1433;da-GAL4/+.* (J and K) Scores of autophagy. Average values were obtained from at least 20 larvae per genotype. Error bar represents standard error of the mean of multiple experiments. (J) In fed conditions, pathogenic Ataxin-3 induces autophagy but normal Ataxin-3 has little effect. (K) In starvation conditions, expression of Ataxin-3, DnaJ-1, and Mrj further induce autophagy; on the contrary, Hsp70 and Imp suppress starvation-induced autophagy. (L–N) Retinal sections. (L) Flies (1 d) expressing SCA3Q84 have some loss of retinal integrity, which is mildly enhanced by reduction of (M) Atg5. (N) Reduction of *atg5* activity alone has normal retinal morphology. Genotypes (L) *w; gmr-GAL4 UAS-SCA3Q84/+*, (M) *UAS-Atg5_IR/UAS-Atg5_IR; gmr-GAL4 UAS-SCA3Q84/+*, and (N) *gmr-GAL4/+; UAS*-*Atg5_IR/UAS-Atg5_IR.* (O) Western blot showing that reduction of Atg5 decreases Ataxin-3 monomer level, concomitant with enhanced toxicity. (P and Q) Cryosections for disease protein (1-d flies, anti-myc). Reduction of autophagy increases disease protein accumulation in the lamina (Q) compared to disease protein alone control (P). Hsc70.K71S also enhances, but it does not cause similar protein accumulations in the retina (unpublished data). Genotype in (P) is same as (L), and (Q) is same as (M). (R and S) Paraffin sections (1-d flies) showing suppression of Ataxin-3 degeneration by Dpld and that Dpld suppression is reduced by limiting autophagy. Genotypes (R) *w; gmr-GAL4 UAS-SCA3trQ78 /JM265* and (S) *UAS-Atg7_IR; gmr-GAL4 UAS-SCA3trQ78/JM265*.

Given that many modifiers mitigated protein accumulation, we asked whether there were interactions with genes of protein degradation pathways. A key pathway thought to modulate the pathogenic polyQ toxicity is the ubiquitin-proteasomal system (UPS) [34]. We therefore tested whether suppression by modifiers that lowered protein levels was dependent upon a fully functional proteasome. Proteasome activity can be reduced by a dominant temperature-sensitive mutation in a proteasome protein subunit (*DTS5*) [35]. In a situation where limiting proteasome activity using this mutation had no effect on SCA3 toxicity on its own, we found that a striking number of modifiers still suppressed polyQ toxicity, thus indicating that they were not sensitive to inhibition of the proteasome by this assay; these included *dpld* alleles (Figure 4; Table 1). In contrast, a striking exception was the class 2 ubiquitin-pathway suppressors: all of these modifiers lost the ability to suppress upon proteasome inhibition with the *DTS* mutation.

### Pathogenic Ataxin-3 Stimulates Autophagy, with Select Modifiers Promoting and Others Preventing Autophagy

Autophagy, or lysosome-mediated protein degradation, has also been implicated in polyQ toxicity and cell survival in situations of stress [36,37]. We therefore asked whether the modifier genes had an effect on this process. First, we determined whether normal or pathogenic Ataxin-3 itself induced lysosomal accumulation reflective of autophagy, by examining the fat body tissue from larvae, a standard assay for autophagy [38]. Normally, well-fed animals show minimal lysosomal induction, whereas starved animals show a dramatic increase, reflected by the uptake of dye (Figure 4F and 4G). Whereas expression of normal Ataxin-3 (SCA3Q27) had minimal effect, expression of pathogenic Ataxin-3-induced autophagy (Figure 4J). To further address the role of autophagy, we determined whether limiting the activity of autophagy genes affected SCA3 toxicity. Key genes to which RNA interference transgenic lines are available include *Atg5*. Whereas reduction of *Atg5* activity on its own had little effect, reducing *Atg5* in the presence of pathogenic SCA3 protein appeared to enhance toxicity, with increased loss of retinal integrity (Figure 4L–4N). This suggests that, normally, autophagy may mitigate toxicity of the pathogenic protein. Reduction of autophagy also enhanced aggregation of the protein by western immunoblot and enhanced cytoplasmic protein accumulation along photoreceptor axons (Figure 4O–4Q).

We then determined whether strong modifier genes also modulated autophagy. We examined two situations: (1) to determine whether strong modifier genes could induce autophagy in well-fed animals when autophagy is normally minimal; (2) to determine whether they could block autophagy under starvation, when autophagy is stimulated as a protective mechanism. We tested these as we considered that a modifier may affect Ataxin-3 pathogenicity either by inducing autophagy-mediated lysosomal degradation of the pathogenic protein, or alternatively, by blocking autophagy if autophagy contributes to loss of the cells in response to mutant polyQ protein. These studies revealed that select modifiers induced, whereas others mitigated, autophagy (Figure 4H, 4I, and 4K). Of the class 1 chaperone modifiers, the two Hsp40 genes (*DnaJ-1^B345.2^* and *mrj ^E1050^*) increased autophagy, whereas Hsp70 and the class 3 modifier *Imp^EP1433^* reduced autophagy. Although Dpld showed no effect in these assays, limiting autophagy by reduction of *Atg5*, *Atg7*, or *Atg12* mitigated Dpld suppression, suggesting that its activity was dependent on autophagy (Figure 4R, 4S, and unpublished data). These and other data (Figures S8 and S9) suggested that Dpld may act upstream of autophagy genes to activate autophagy in select situations. We also tested available GFP protein trap lines to examine localization of modifier proteins. Although none of these lines showed GFP immunostaining, one line with a protein trap insertion in *Hsc70Cb* enhanced SCA3 neuronal toxicity and increased protein accumulation in the neurophil similar to autophagy genes (Figure S8G–S8K). Taken together, these studies indicated that the modifiers, despite broad molecular nature, mitigated situations of protein misfolding; in some cases their activity appeared dependent on the proteasome, whereas others may involve autophagy-based protein clearance or autophagy-related cell loss.

### Select Modifiers Affect Other Pathogenic Human Disease Models

**Figure 5:**
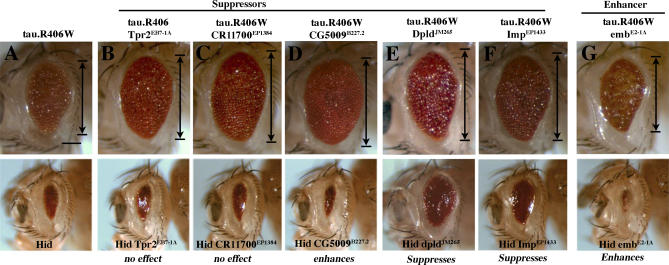
Select Ataxin-3 Modifiers also Modulate Tau Toxicity (A) Top: expression of pathogenic human tau.R406W causes a rough and reduced eye [41]. Genotype *w; gmr-gal4/+; UAS-tau.R406W/+.* Bottom: Ectopic expression of apoptotic gene *hid* ablates the eye. Genotype *w; gmr-GAL4 gmr-hid /+*. Genes shown modulated tau.wt and tau.R406W similarly. (B–D) *Tpr2^EB7-1A^* (B), polyubiquitin *CR11700^EP1384^* (C), and *CG5009^B227.2^* (D) suppress pathogenic tau. *Tpr2^EB7-1A^* (B) and *CR11700^EP1384^* (C) have no effect on Hid-induced programmed cell death. Genotypes (upper panel B) *w; gmr-GAL4/+; UAS-tau.R406W* /*EB7-1A*, *(C) EP1384; gmr-GAL4/+; UAS-tau.R406W* /+, and *w; gmr-gal4/B227.2; UAS-tau.R406W* /+. (Lower panel, B) *w; gmr-GAL4 gmr-hid* /*EB7-1A, (C) EP1384; gmr-GAL4 gmr-hid* /+, and (D) *w; gmr-GAL4 gmr-hid/B227.2*. (E–G) *dpld^JM265^* (E), *Imp^EP1433^* (F), and *emb^E2-1A^* (G) modulate tau toxicity and programmed cell death in a similar manner, suggesting that their effect on tau may be due to modulation of cell death. Genotypes (upper panel, E) *w; gmr-GAL4/+; UAS-tau.R406W* /*JM265*, (F) *EP1433; gmr-GAL4/+; UAS-tau.R406W* /+, and (G) *w; gmr-GAL4/E2-1A; UAS-tau.R406W* /+. (Lower panel, E) *w; gmr-GAL4 gmr-hid* /*JM265*, (F) *EP1433; gmr-GAL4 gmr-hid* /+, and (G) *w; gmr-GAL4 gmr-hid/E2-1A*.

The modifiers were selected based on ability to modulate SCA3 degeneration; however, our studies suggested that the modifiers may have broader functions in protein misfolding. We therefore determined whether they could modulate toxicity of tau. Abnormal accumulation of tau in neurofibrillary tangles or mutations in tau are associated with Alzheimer disease and frontotemporal dementia [39]. Tau-induced degeneration is mitigated by the caspase inhibitor P35 and DIAPs, implicating programmed cell death pathways in tau toxicity [40]. Expression of normal (tau.wt) or mutant (tau.R406) tau causes toxicity reflected in a reduced and degenerate eye [41]. Co-expression of the class 1 chaperone *Tpr2^EB7-1A^* and the *Hsc70Cb* line class 2 modifier polyubiquitin *CR11700^EP1384^*, and class 3 modifiers *dpld ^JM265^*, *Imp^EP1433^*, and *CG5009^B227.2^* strikingly suppressed toxicity of tau (Figure 5; Figure S8). The class 3 modifier *emb^E2-1A^* enhanced tau (Figure 5), whereas *NFAT ^EP1335^* enhanced tau.wt, but had no effect on mutant tau.R406W (unpublished data). We then examined the ability of the modifiers to affect programmed cell death. Several class 3 modifiers had an effect on *hid*-induced eye loss: co-expression of *emb^E2-1A^* and *NFAT^EP1508^* enhanced *hid*, whereas *Imp^EP1433^* and *dpld^JM265^* alleles suppressed *hid*-induced cell death (Figure 5 and unpublished data). Alleles of those genes that modulated tau and programmed cell death similarly (*emb*, *dpld*, *NFAT*, and *Imp*) may modulate tau toxicity by altering cell death. In contrast, the others (*Tpr2*, polyubiquitin *CR11700,* and *Hsc70Cb*) likely modulate tau toxicity through other means. Taken together, these data indicate that select modifiers that influence cell survival and protein misfolding may be common to both SCA3 and tau-induced degeneration.

## Discussion

We present a detailed functional analysis of genetic modifiers of pathogenic Ataxin-3 induced neuronal toxicity. The majority of modifiers fell into chaperone and ubiquitin pathways, consistent with the proposed function of Ataxin-3 in the ubiquitin-proteasome pathway, as well as pathways implicated in polyQ toxicity. Strikingly, however, biological assays for the activity of modifiers, some of which are predicted to function in diverse biological processes, indicated that their activities also converge on protein misfolding. Several protein quality control pathways appear involved, including the UPS and autophagy. These findings underscore the significance of protein quality control pathways to SCA3 and potentially other age-dependent protein conformation diseases.

### A Genetic Screen for Modifiers That Mitigate PolyQ Degeneration

Our screen was designed to identify upregulation modifiers, targeting genes whose increased activity could modulate neuronal toxicity and degeneration of a pathogenic Ataxin-3 protein. This screen identified activities that may become compromised or normally insufficient in the disease protein situation. A number of modifiers appeared dosage sensitive, in that reduction of the genetic region encoding the gene enhanced degeneration. This suggests that, at least for select modifiers, their normal activity is also critical for maintenance of neurons in disease.

Because of the severity of the degenerate eye, we may have selected for particularly strong modifiers. Modifiers were also initially selected for the ability to mitigate an external eye degeneration, rather than direct neuronal toxicity. Despite this, all of the modifiers mitigated internal neural degeneration. This indicates that the degree of external eye degeneration is a reasonable predictor of internal neuronal integrity. A number of modifiers were identified multiple times (*dpld*, *NFAT,* *emb,* and *dbr*), although most were found only once, indicating that the screen is not saturated. Additional screens using different mutagens or different types of screening approaches may reveal additional and different classes of modifiers. Although the modifiers were selected for mitigation of toxicity of a truncated Ataxin-3 protein, nearly all effectively mitigated toxicity of the full-length pathogenic protein. Our previous and other studies suggest that truncation may normally occur in disease to remove the N-terminal ubiquitin protease domain and would dramatically enhance degeneration [15,29,30]. Therefore, these are efficacious modifiers that mitigate toxicity of various forms of the pathogenic protein.

### Chaperones and Ubiquitin-Pathway Components Are Critical Regulators of SCA3

**Figure 6:**
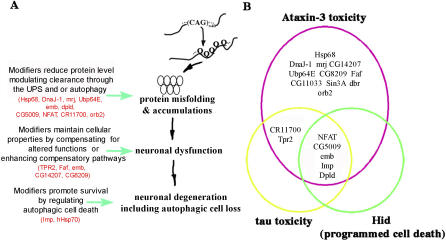
Activities of Modifier Genes of Ataxin-3-Associated Neurodegeneration (A) Modifiers of pathogenic Ataxin-3 toxicity may (1) reduce disease protein accumulation into NI in a manner sensitive to proteasome activity and/or by modulating autophagy, (2) promote cellular functionality in situations of misfolded protein, and/or (3) promote neuronal survival by regulating autophagic cell loss. (B) Select modifiers of Ataxin-3 toxicity reveal genetic links to tau toxicity and Hid-induced programmed cell death. *Tpr2^EB7-1A^*, polyubiquitin *CR11700^EP1384^*, and *CG5009^B227.2^* suppress both Ataxin-3 and tau toxicity, implicating chaperone and ubiquitin proteasome activity as modifiers of tau effects. Modifiers with similar effects on tau and hid-induced cell death may be modulating tau by modulating cell death; however, these modifiers likely act in a distinct way on Ataxin-3-associated degeneration, which is not sensitive to programmed cell death genes but rather appears modulated by the microRNA *bantam* [22].

The majority of modifiers were either chaperones or, by sequence and functional tests, modulated ubiquitin-mediated protein quality control pathways. Whereas those that have sequence implications in this pathway might be anticipated, a surprise was that many genes with widely divergent molecular activities were functionally implicated in this process. These data suggest that many genes can modulate situations of protein misfolding, and moreover, suggest that protein misfolding is central to polyQ pathogenesis. It is also possible that, given the normal function of Ataxin-3 in ubiquitin-modulated pathways, the screen selected for modifiers that impinge on normal activities of Ataxin-3. Among the modifiers, some decreased protein accumulation, whereas others suppressed with no apparent change in Ataxin-3 inclusions. Of those that decreased Ataxin-3 accumulation, some appeared dependent on functional proteasome activity, whereas others stimulated autophagy, or both, implicating various mechanisms that attenuate Ataxin-3 degeneration. Among the modifiers was polyubiquitin, arguing that the level of ubiquitin itself may normally be insufficient to handle the misfolded protein over prolonged periods. This is consistent with the lack of a robust stress response to pathogenic polyQ [42]. In a Caenorhabditis elegans of model of polyQ, Gidalevitz et al. [43] reveal that polyQ expansions can cause temperature-sensitive alleles of various unrelated genes (*paramyosin*, *dynamin*, and *ras*) to display the mutant effect at what would normally be permissive conditions. This finding suggests that long polyQ runs can cause a general disruption of the cellular protein-folding environment to affect the temperature-sensitive mutant proteins. Our work used a pathogenic human disease protein Ataxin-3 and revealed that many modifiers that mitigate Ataxin-3 toxicity also modulate general protein misfolding. Taken together, these results suggest that, in the absence of upregulation of the various components needed, the cell may become overwhelmed, triggering deleterious effects [32]. Our detailed analysis of modifiers of Ataxin-3 have revealed a variety of biological processes that can help manage pathogenic polyQ protein, as well as specific genes of interest (Figure 6A).

The precise molecular pathways by which those modifiers that are not clearly within protein folding pathways can modulate misfolding to affect neurodegeneration requires further study. We envisage, however, that they function by their predicted molecular mechanisms, but on targets that impinge on cellular protein homeostasis. For example, chromatin modifiers may tweak the expression of a variety of genes in such processes, whereas translational regulators may translationally modify such genes. The finding that a variety of cellular functions are involved would be consistent with RNA interference screens in C. elegans that identified a variety of genes that impinge on cellular protein homeostasis as enhancers of the aggregation of polyQ protein [20]. These findings, along with other studies including those we present here, underscore the critical importance of proper protein homeostasis to most—if not all—cellular functions, such that a variety of genes can influence this process.

Among our modifiers, some that might have been expected to act in a similar manner, appeared to have distinct biological effects*.* The class 2 modifiers showed complete dependence on proper proteasome activity, while other modifiers, including alleles of DnaJ-1 and Imp, appeared to be insensitive to limiting proteasome activity, but rather affected autophagy in stress conditions. Hsp70 suppresses multiple stress conditions including protein misfolding, starvation-induced autophagy, and paraquat-induced oxidative stress [11], suggesting it may both facilitate UPS activity, but also block autophagy-dependent degeneration. In vivo, these pathways interplay to maintain proper neuronal function in the face of disease, thus further study of the modifiers may allow greater molecular identification of the individual pathways, as well as their integration.

Although only select modifiers affected protein accumulation as assayed by immunohistochemistry, all affected the solubility of the disease protein by biochemical analysis. The relationship between protein inclusions and proposed toxic oligomers is still under investigation, but it seems reasonable to suggest that the change in solubility may reflect activity of the modifiers to buffer or alter toxic conformations of the protein. Our efforts to detect toxic oligomers of Ataxin-3 using available antibodies [33,44,45] are still in progress. We note that, despite gross similarity, SCA3 degeneration is not identical to general misfolding: some suppressors of Ataxin-3 toxicity strikingly enhanced dominant-negative Hsp70 (upregulation of DnaJ-1 and Tpr2), whereas the enhancer *CG11033^E3093^* suppressed it.

The UPS and autophagy pathways function in either degeneration or polyQ protein pathogenicity [46]. Our findings indicate that these pathways at least in part function in the removal or decrease of the toxic protein, thereby reducing degeneration. It is also possible that activities of these pathways may be inhibited during pathogenesis; such inhibition of normal activity would contribute to disease pathology [47–49]. The normal function of Ataxin-3 is in ubiquitin-modulated pathways [23–26]; our data suggest the possibility that Ataxin-3 may also modulate autophagy. Normal physiological levels of autophagy are critical to integrity of neurons, as loss of autophagy causes degeneration associated with ubiquitinated inclusions [50,51]. Our studies also reveal that compromise of autophagy pathways strikingly increased cytoplasmic Ataxin-3 accumulations in the neurophil without an obvious change in NIs. This may indicate that autophagy normally modulates cytoplasmic accumulation of the disease protein. Previous studies show that cytoplasmic polyQ protein is very toxic and blocks axonal transport [52]. These findings suggest that perturbations in autophagy may enhance cytoplasmic toxicity further.

In addition to regulation of the disease protein level and misfolding, results with select modifiers (Hsp70 and Imp) suggest that autophagy may also regulate loss of the cells. Given that our previous findings failed to reveal a clear role of caspase-dependent cell death in SCA3 [22], autophagy may be a mechanism of cell loss in this situation. Identification of other components of UPS and autophagy pathway will give further insights into how the disease protein is degraded and mechanisms of neuronal loss.

### Relationship to Other Modifier Pathways and Screens

Genetic screens have been performed for modifiers of Ataxin-1 [19] and of pure polyQ domains [18], revealing similar components of protein folding and degradation: DnaJ-1 [18,19] and Tpr2 [18], but also RNA binding proteins and transcription factors. Although components of ubiquitin pathways that modulate Ataxin-1 appear distinct from Ataxin-3, this may reflect different regulators required for modifying different proteins or lack of saturation of the screens. Similarly several RNA binding proteins identified as modifiers of Ataxin-1 were suggested to reflect Ataxin-1 function as a putative RNA binding protein; our data suggest that it is also possible that these modifiers work more fundamentally to modulate protein solubility or levels. Our other work, however, has revealed a role for microRNAs in modulating neuronal survival in neurodegenerative situations [22,53], which has recently been extended to vertebrate neuronal integrity as well [54]. A C. elegans screen revealed a large number of modifiers of a pure polyQ aggregation phenotype [20]. Several modifiers identified in that screen affect RNA synthesis, processing, and protein synthesis as modifiers of misfolding. It will be important to test these modifiers against various specific disease proteins, as the action of a pure polyQ repeat may be distinct from a pathogenic repeat within a host protein.

One reason for global commonality among modifiers of polyQ disease proteins may be that the proteins themselves fall within a common interacting protein network. Recent studies using the yeast two-hybrid approach and proteomic databases reveal a protein interaction network of the ataxia proteins [55]. A surprise was that many different ataxia proteins, including Ataxin-3, fall within a few interaction steps from one another, suggesting that their common phenotypes may be a reflection of common interactions, which, when perturbed, contribute to disease. In that study [55], seven proteins were identified as direct interaction partners of Ataxin-3; however none of these appeared in our screen. Another direct binding protein–VCP [56]—was also not identified. However, the human orthologues of the majority of the genetic modifiers we defined fit into the protein interaction network at various points; some are direct interactors of other ataxia disease proteins, and others are one or more steps removed (Table S3).

Alzheimer disease and polyQ diseases are two unrelated human neurodegenerative diseases that cause neuronal degeneration in distinct brain regions [1,2,57]. Therefore, no overlap was expected between modifiers of these disease proteins in flies; indeed previous studies suggest minimal overlapping genes [58]. Surprisingly, we found that select modifiers of Ataxin-3 suppressed tau-degeneration, including the cochaperone Tpr2 and polyubiquitin (Figure 6B). This finding is consistent with cell culture studies and C. elegans RNA interference screens that implicate chaperones as modifiers of tau degeneration [59,60]. Further study of these modifiers, especially those that may be in common among different disease proteins, should provide the foundation for new therapeutic insight.

## Materials and Methods

### Drosophila stocks and crosses.

Fly lines were grown in standard cornmeal molasses agar with dry yeast at 25 °C. Transgenic lines *UAS-SCA3trQ78*, *UAS-SCAtrQ61, UAS-SCA3Q78,* and *UAS-SCA3Q84* are described [14–16]. General stock lines, deficiency lines, and specific alleles of the EP modifiers were obtained from the Bloomington Drosophila stock center. The *emb* mutant lines were from C. Samakovlis (Wenner-Gren Institute Stockholm University, Stockholm, Sweden) [61]. *rh1-GAL4*, *gmr-hid*, UAS-DTS5, autophagy inverted repeat lines (*UAS-Atg5_IR* and *UAS-Atg7_IR*), tau transgenic lines (*UAS-htau* and *UAS-htau.R406W*), *UAS-brat*, and Hsc70C protein trap line were kindly provided by C. Desplan (New York University, New York, New York, United States), H. Steller (Rockefeller University, New York, New York, US), J.M. Belote (Syracuse University, Syracuse, New York, US), T. Neufeld (University of Minnesota, Minneapolis, Minnesota, US), M. Feany (Harvard Medical School, Boston, Massachusetts, US), R. Wharton (Duke University Medical Center, Durham, North Carolina, US), M. Buszczakl (Johns Hopkins University, Baltimore, Maryland, US), respectively. The dominant-negative Hsp70 line is described [32]. *UAS-dpld* FLAG, untagged, and gfp fusion lines were generated from cDNA clone LD02463 by subcloning into the pUAST vector [62]. Excision lines for reversion were made by crossing the *EP* insertions to lines bearing transposase, then screening for loss of the *EP* element.

For the genetic screen, virgin females of the starter line *EP55*, bearing an *EP* insertion on the X chromosome [27], was crossed to males with transposase. Males were selected and crossed to virgin females that expressed the disease gene (*w/w*; *gmr-GAL4 UAS-SCA3trQ78 Tft*/*CyO*). F_1_ progeny were screened for males with either suppressed or enhanced eye phenotypes as compared to controls; any modifiers were then outcrossed and balanced. EP insertions were confirmed to be single insertions through outcrossing, as well as plasmid rescue.

### Histochemistry.

Epon sections, paraffin sections, and cryosections of adult heads were performed as described [14,16]. Primary antibodies for immunostaining were anti-HA primary antibody (Y-11, 1:50, Santa Cruz Biotechnology; and 12CA5, 1:100, Roche), anti-Myc (9E10, 1:100, Santa Cruz Biotechnology), anti-Hsp70 (7FB, 1:1,000,) [63], and anti-Gfp (A6455, 1:50, Molecular Probes). Secondary antibodies included anti-mouse or anti-rabbit conjugated to Alexa Fluor 594 or 488 (1:200 or 1:100, Molecular Probes). Western immunoblots were performed as described [16]. Primary antibodies used were HA-HRP (3F10, 1:500, Roche), rat anti-Hsp70 (7FB, 1:2,000), and mouse anti-tubulin (E7, 1:2,000, Developmental Studies Hybridoma Bank), with the secondary goat anti-rat IgG (1:2,000, Roche) and goat anti-mouse IgG (1:2,000, Chemicon International).

### Molecular biology.

To define the sites of *EP* insertion, plasmid rescue was performed [27]. To confirm single hits in the genome, multiples of clones from each *EP* line were sequenced with an *EP* 3′ P-end specific primer (5′-CAA TCA TAT CGC TGT CTC ACT CA-3′). The flanking DNA sequence was used to query flybase BLAST to define the nearby gene and exact site of insertion. Upregulation of the genes by the *EP* element was confirmed by northern analysis, comparing the *EP* line alone, to wild-type fly controls with the *EP* line in the presence of a GAL4 driver, using gene-specific probes generated by reverse-transcription PCR using an EP-specific primer and gene-specific primers to the target genes. That the *EP* insertions were single insertions was independently verified genetically by molecular and phenotypic analysis of lines.

The effect of the modifiers on transgene expression levels was examined by real-time PCR (see Text S1 and Figure S2) and by reverse-transcription PCR. For the latter, total RNA was extracted with the RNAeasy kit (74104, QIAGEN) following manufacturer's instructions. cDNA synthesis was done using SuperScript First Strand Synthesis for reverse-transcription PCR (12371–019, Invitrogen). PCR was performed using primers: for the SCA3 transcript, 5′-CTATCAGGACAGAGTTCACAT-3′ (forward) and 5′-CAGATAAAGTGTGAAGGTAGC-3′ (reverse); for the GAL4 transcript, 5′-GTCTTCTATCGAACAAGCATGCGA-3′ (forward) and 5′-TGACCTTTGTTACTACTCTCTTCC-3′ (reverse) and for rp49 control, 5′-CCAGTCGGATCGATATGCTAA-3′ (forward) and 5′-ACCGTTGGGGTTGGTGAG-3′ (reverse).

### Behavioral assays.

Phototaxis was performed as described [15]. The percentage of flies in the light and dark chambers represent an average of three independent groups of flies. At least 100 flies were tested for each genotype, 20 flies were used in each experiment.

### Autophagy assays.

Third instar larval fat body tissues were stained with Lyso Tracker Red DND-99 (L-7528, Molecular Probes) and Hoechst (H3570, Molecular Probes) as described [38]. For each genotype, well-fed and starved larvae were used as negative and positive controls for the assay conditions. A score of 4 was given to control larvae grown under starvation condition; autophagy for other genotypes was scored relative to this. Controls (driver line alone and/or modifier alone, in starvation and well-fed conditions) were performed for each modifier genes in parallel to the experimental situation. The final autophagy score represents the average of 20 larvae from each genotype.

## Supporting Information

Figure S1 Modifiers of SCA3trQ78-Associated Neurodegeneration External eye and internal retinal sections of 1-d flies.
(A) Control external and internal eye. Normal retinal thickness is indicated with yellow arrow. Genotype *w; gmr-GAL4/+.*
(B) Expression of strong SCA3trQ78 in the eye causes external and internal degeneration with severe pigment loss and markedly reduced retinal thickness. Genotype *w; gmr-GAL4 UAS-SCA3trQ78/+*.
(C–T) Suppressors of SCA3tr-Q78 associated neurodegeneration in *trans* to *w; gmr-gal4 UAS-SCA3trQ78. EP* lines are (C) *E407*, (D) *B345.2*, (E) *E1050*, (F) *EP1348*, (G) *EB7-1A*, (H) *EP1384*, (I) *E213-1A*, (J) *B3-Sa*, (K) *E659*, (M) *E2-1A*, (N) *B9-E*, (O) *EP1335*, (P) *EP456*, (Q) *JM265*, (R) *B8-S*, (S) *EP1433* and (T) *B227.2*. (L) *EP3093* enhances SCA3trQ78-associated neurodegeneration and lethality.
(1.9 MB JPG)

Figure S2 Control Experiments for SCA3 Modifiers (A) (Top) Western analysis for SCA3trQ78 protein level (detected with anti-HA) in 1-d fly heads. Experiments were performed using the late onset gene expression driver *rh1-GAL4*. At 1 d, the pathogenic protein is soluble by immunocytochemistry and western analysis. The Ataxin-3 protein level was similar in the various situations, indicating that the modifiers do not affect expression from the GAL4-UAS system. Genotypes: *w; UAS-SCA3trQ78; rh1-GAL4* in *trans to +* or the various *EP* modifier lines as indicated.
(A) (Bottom) Real-time PCR for level of transgenes shown are *Tpr2^EB7-1A^, Faf^E659^, Sin3A^B9-E^, dbr^EP456^, dpld^JM265^, orb2^B8-S^, Imp^EP1433^; CG5009^B227.2^* was confirmed by reverse transcription PCR (unpublished data). Genotypes: *w; UAS-SCA3trQ78; rh1-GAL4* in trans to *+* or *EP* modifier lines indicated.
(B–F) Examples of modifiers that failed to affect tau toxicity. All modifiers were tested for ability to modulate the deleterious effects of a number of additional genes, including tau, hid, and others (below). Although select modifiers affected tau toxicity (either to enhance or suppress, see Figure 5), others had no effect. The inability to modulate such additional gene activities was additional evidence that the modifiers did not affect the GAL4-UAS system. Tau-induced degenerate eye is not modulated upon co-expression of *EP* alleles of (C) *Hsp68*, (D) Ubp64E, (E) CG11103, or (F) Sin3A. Genotypes: (B) *w; gmr-GAL4/+; UAS-tau.R406W/+* , (C) *w; gmr-GAL4/+; UAS-tau.R406W/ E407*, (D) *w; gmr-GAL4/+; UAS-tau.R406W/ E213-1A*, (E) *w; gmr-GAL4/+; UAS-tau.R406W/EP3093*, and (F) *w; gmr-GAL4/+; UAS-tau.R406W/ B9-E.*
(G–K) Detailed analysis of controls for alleles of the *dpld* gene.
(G) Western analysis for SCA3trQ78 protein expression in 1-d fly heads. The *EP* allele of *dpld* (*dpld^JM265^*), generated transgenic lines of *dpld* (*UAS-dpld, dpld^J990^*), and the precise excision line of the *dpld ^JM265^* allele (*dpld ^JM265-ex^*) showed similar levels of disease protein at early time points compared to the control (+). This indicated that upregulation of *dpld* did not affect expression from the GAL4-UAS system; rather further studies showed that Dpld affected long-term accumulation of the protein (see Figure 3F and 3G). Genotypes: lane 1, *w;* UAS-*SCA3trQ78/+; rh1-GAL/+*; lane 2, *w; UAS-SCA3trQ78/dpld ^JM265^; rh1-GAL4/+*; lane 3, *w; UAS-SCA3trQ78/+; rh1-GAL4/UAS-dpld^J990^*; lane 4*, w; UAS-SCA3trQ78/dpld ^JM265-ex^; rh1-GAL4/+.*
(H–K) Effect of *dpld* expression on various deleterious GAL4/UAS-induced eye phenotypes.
(H and I) *dpld* expression had no effect on *eyeless*-induced disruption of the eye. Genotypes: (H) *w; ey-GAL4 UAS-eyeless/ dpld ^JM265^* and (I) *w/+; ey-GAL4 UAS-eyeless/+.*
(J and K) *dpld* expression did not affect the rough eye phenotype induced by expression of MAP2. Genotypes: (J) *w; gmr-GAL4 UAS-MAP2/ dpld ^JM265^* and (K) *w/+; gmr-GAL4 UAS-MAP2/+.*
(1.0 MB JPG)

Figure S3 Conserved Domains of Modifier Genes The modifiers fall into three major classes based on conserved sequence domains: genes involved in chaperone pathways, in ubiquitin pathways, and miscellaneous functions. The conserved domains for the first category include Hsp70, DnaJ, DNAJ-C, Hsp20, and tetratricopeptide repeat (TPR). The second category has ubiquitin, ubiquitin carboxy hydrolase (UCH), ubiquitin associated (UBA), ubiquitin regulatory (UBX), and F-box domains. The conserved domains for the third category include Importin-beta N terminus (IBNN), paired amphipathic helix repeat (PAH), rel homology domain (RHD), transcription factor/cell surface receptor immunoglobulin-like fold (TIG), RNA recognition motif (RRM), KH, and acyl-CoA oxidase (ACOX) domain.
(665 KB JPG)

Figure S4 Ataxin-3 Modifier Genes Mitigate Toxicity of PolyQ Protein in the Nervous System (A) Animals expressing SCA3trQ78 in the nervous system do not develop to adults, with lethality of larvae and pupae that fail to form completely (pupa shown).
(B) With co-expression of *EP1050*, now the animals develop to the pharate pupal stage, with eyes, wings, and legs fully formed, though the animals fail to emerge from the pupal case. Genotypes: (A) *elav-GAL4; UAS-SCA3trQ78(S)/+* and (B) *elav-GAL4; UAS-SCA3trQ78(S)/EP1050.*
(C) Table of lethality with or without added select modifier genes. *, Lethality assessed with neuronal expression by the *elav-GAL4* driver, which is expressed in all neurons through development and in the adult.
(901 KB JPG)

Figure S5 Genomic Organization of *dpld*
(A) Genomic organization of *dpld* with insertion sites of EP alleles indicated. The A form of the *dpld* transcript is shown (three splice variants are predicted), black represents exons, and blue indicates the 5′ UTR and 3′ UTR. EP insertions are in the same orientation as gene expression. *dpld* is on 2R, cytological position 43C5. The centromere is to the right. Restriction enzyme sites are: X, XbaI; H, HindIII; R, EcoRI; B, BamHI; S, SacII; P, PstI.
(B) Schematic representation of Dpld protein and select homologs (Dm Brat, Ce Lin-41, and Homo sapiens Lin-41). Blue is the B-box zinc finger, yellow is the coiled-coil, red is NHL repeats, and pink is a ring finger domain. The percent identity of the NHL domain to that of Dpld is noted.
(C) NHL domain based evolutionary tree showing relatedness of Dpld to that of other RBCC-NHL family members in nematode, fly, and human. Dpld is most homologous to Lin-41; the other studied fly NHL protein Brat is distantly related.
(D) Sequence alignment of the B1 and B2 boxes, and the NHL domain of Dpld to those of Brat, human Lin-41, and C. elegans Lin-41. Amino acid identities in black; similarities and identities in three of the four sequences are in gray. The cysteine and histidine residues that define the B-box subclass of Zn finger are marked with an asterisk (*). The positions of NHL repeats are noted.
(806 KB JPG)

Figure S6 Dpld Suppresses Ataxin-3-Associated Neurodegeneration External eye and internal retinal sections of 1-d flies.
(A) Control fly bearing only the driver line. Genotype *w; gmr-GAL4/+*.
(B) Ataxin-3 induces severe retinal degeneration. Genotype *w; gmr-GAL4 UAS-SCA3trQ78*/+.
(C) Co-expression of *dpld^JM265^* with SCA3trQ78 results in normal pigmentation and improved retinal structure. Genotype *w; gmr-GAL4 UAS-SCA3trQ78/dpld^JM265^.*
(D) Expression of *dpld* from a *UAS-dpld* transgene improves retinal structure, indicating that suppression of polyQ toxicity is due to increased Dpld activity. Genotype *w; gmr-GAL4 UAS-SCA3trQ78 /+; UAS-dpld ^J990-P2^/+.*
(E–G) Tangential retinal sections of fly expressing SCA3trQ78 alone or with Dpld. Flies expressing SCA3trQ78 have a normal retina (E) at 1 d, which undergoes severe progressive degeneration by (F) 21 d. Genotype *w; UAS-SCA3trQ78/+; rh1-GAL4/+*.
(G) Flies with SCA3trQ78 and UAS-dpld^J990-P2^ are normal. Genotype *UAS-SCA3trQ78/+; rh1-GAL4 /UAS-dpld.*
(H) Rhabdomere counts in individual ommatidia by optical neutralized corneal pseudopupil (see reference contained in Text S1). Flies expressing SCA3trQ78 show degeneration in the number of rhabdomeres per ommatidial unit at 21 d. Flies expressing SCA3trQ78 and *dpld^J990-P2^* retain rhabdomere numbers over 21 d; dpld^JM265^ has partial rescue compared to controls.
(1.5 MB JPG)

Figure S7 Select Modifiers More Effectively Mitigate Toxicity of Full Length Ataxin-3 (A) Expression of full-length SCA3Q84 causes a mild eye degeneration. Genotype *w; gmr-GAL4 UAS-SCA3Q84 /+*.
(B–C) The modifiers (B) *CG14207^EP1348^* and (C) polyubiquitin *CR11700* ^*EP1384*^ suppress full-length disease toxicity stronger than they do the truncated disease protein. Genotypes *w; gmr-GAL4 UAS-SCA3Q84* in trans to *EP1348* and *EP1384*.
(482 KB JPG)

Figure S8 Additional Details of Dpld and Hsc70C (A–C) Horizontal eye section of a fly eye expressing *dpld-eYFP* shows (B) nonhomogenous distribution and localization to membranous structures. (A) Hoechst staining. (C) Overlay shows that Dpld does not localize to nucleus but is concentrated around it. Genotype *w; UAS-dpld-eYFP* in trans to *gmr-GAL4*.
(D–F) Horizontal retinal section showing Dpld localization in the presence of SCA3trQ78. (D) eYFP staining for Dpld and (E) HA staining for SCA3trQ78. (F) Overlay shows that Dpld is not recruited to the SCA3trQ78 aggregates. Genotype *w; gmr-GAL4 UAS-SCA3trQ78* in trans to *UAS-dpld-eYFP.*
(G and H) GFP insertion allele of *Hsc70Cb* enhances Ataxin-3 neurotoxicity. Hsc70CbGFP fusion protein was confirmed by Western immunoblot (unpublished data). The line is homozygous lethal indicating a potential loss of function of allele of *Hsc70Cb*. Genotypes *w; gmr-GAL4 UAS-SCA3trQ78/+* (H) and *w; gmr-GAL4 UAS-SCA3trQ78/+; Hsc70Cb^CB02656^/+.*
(I–K) Hsc70Cb protein trap line strikingly increases Ataxin-3 inclusions in the lamina and medulla, indicating enhanced cytoplasmic disease protein. (I) Hoescht staining for nuclei. Genotypes *gmr-GAL4 UAS-SCA3Q84/+* (I and J), *w; gmr-GAL4 UAS-SCA3Q84/+; Hsc70Cb^CB02656^/+.*
(L–O) *Hsc70Cb* suppresses (L and M) normal and (N and O) mutant R406W tau-induced degeneration. Genotypes (L) *w; gmr-GAL4/+; UAS-tau/+*, (N) *w; gmr-GAL4/+; UAS-tau/ Hsc70Cb^CB02656^*, (M) *w; gmr-GAL4/+; UAS-tau.R406W/+*, and (N) *w; gmr-GAL4/+; UAS-tau.R406W/ Hsc70Cb^CB02656^*.
(2.1 MB JPG)

Figure S9 Dpld Modulates Body Size (A–F) *dpld* and *brat* share a function in growth regulation (see reference contained in Text S1), but the activities of *dpld* to suppress polyQ toxicity and cell death are special to *dpld*. The effect of *dpld* on eye and body size were mitigated by reducing autophagy gene activity (not shown).
(A right, and C) Dpld expression during development reduces body size (A, fly on the right) and (C) eye size, similar to *brat* over-expression. Genotype in (A) *w; act5C-GAL4* in trans to + or *UAS-dpld* and (C) *w; ey-GAL4/UAS-dpld.*
(A left, B) Normal fly body and eye size. Genotype (A) *w; act5C-GAL4/+* and (B) *w; ey-GAL4/+*.
(D, E) Directed expression of *brat* does not mitigate degeneration induced by SCA3trQ78 either (D) externally or (E) internally. Genotype *w; gmr-GAL4 UAS-SCA3trQ78* in trans to *UAS-brat*.
(F) Brat does not mitigate *hid*-induced programmed cell death. Genotype *w; gmr-hid gmr-GAL4* in *trans* to *UAS-brat*.
(769 KB JPG)

Table S1 Summary of Genetic Modifiers of Ataxin-3 The location and orientation of the *EP* insertions are indicated, including the method used to confirm that the insertions caused overexpression of the targeted gene. Note that insertions into novel genes were reverted to confirm that the EP insertion was causal in modification.
(98 KB DOC)

Table S2 Loss-of-Function Deficiency Regions and Alleles of Modifiers of Ataxin-3 Neurodegeneration That Were Tested Alleles and deficiency lines of Ataxin-3 modifiers were tested for modulation of polyQ toxicity. See also Figure 1K–1O.
(89 KB DOC)

Table S3 Protein Interactions of Ataxin-3 Genetic Modifiers List of human orthologues of the fly modifiers found. The degree of interaction from Ataxin-3 is from Table 5 of Lim et al. (see reference contained in Text S1). Degree of interaction is based on the distance each orthologue is from the seven primary interactors defined for Ataxin-3 (ARHGAP19, EWSR1, LIN10, RAD23A, RAD23B, TEX11, and PRKCABP) (see reference contained in Text S1) and VCP/TER94 (see reference contained in Text S1).
(122 KB DOC)

Text S1 Supplemental Methods (62 KB DOC)

### Accession Numbers

The accession numbers of the proteins used in the analysis in Figure S5 from the National Center for Biotechnology Information (NCBI) (http://www.ncbi.nlm.nih.gov) are Dm Dappled (NM_165533), DmCG15105 (NM_137546), Dm Brat (NM_057597), Dm Mei-P26 (NM_143765), Ce Lin-41 (NM_060086), Hs Lin-41 (XM_067369), Hs TRIM2 (NM_015271), Hs TRIM3 (NM_006458), and Hs TRIM32 (NM_012210).

## Abbreviations

GFP: green fluorescent protein
NI: nuclear inclusion
polyQ: polyglutamine
SCA3: spinocerebellar ataxia type-3
UPS: ubiquitin-proteasomal system
