# Supplementary material for: Genome-Wide Screen for Modifiers of Ataxin-3 Neurodegeneration in Drosophila
Source: PLoS Genet. 2007 Oct 19;3(10):e177. doi: 10.1371/journal.pgen.0030177 (PMC2041992; doi:10.1371/journal.pgen.0030177)
Supplement: Supplementary file 1 [file pgen.0030177.sd001.doc]

Supplemental Data to:

# Genome-wide screen for modifiers of Ataxin-3 neurodegeneration in *Drosophila*

Julide Bilen^1, 3^ and Nancy M. Bonini^1,2 *^

^1^Department of Biology

University of Pennsylvania

^2^Howard Hughes Medical Institute

Philadelphia, PA 19104-6018

*Direct correspondence to:

Dr. Nancy M. Bonini

Department of Biology

415 S. University Avenue

University of Pennsylvania, Philadelphia, PA 19104-6018

Phone: 215-573-9267

FAX: 215-573-5754

Email: [nbonini@sas.upenn.edu](mailto:nbonini@sas.upenn.edu)

^3^Current Address:

Howard Hughes Medical Institute

Janelia Farm Research Campus

19700 Helix Drive

Ashburn VA 20147

**Supplemental Experimental Procedures**

**Molecular Biology**

To confirm the GAL4-dependent expression of the genes adjacent to each *EP* insertion, the lines were crossed to *gmr-GAL4* and controls. Total RNA was isolated from the heads using Trizol based extraction. Then the expression level of each gene was assayed by Northern with a probe specific to modifier gene sequence. For some of the EPs, we used UAS specific upstream primer and gene specific downstream primer to confirm the GAL4 dependent over-expression of the line by RT-PCR. RNA was prepared from heads of animals of the appropriate genotypes as described in Methods and Materials. 600 ng RNA was treated with DNase following manufacturer protocols (Ambion/Applied biosystems, Cat # AM2238). 240 ng of RNA equivalent of cDNA was reverse transcribed using random hexamer primers following manufacturer’s instructions (SuperScript First-Strand Synthesis Kit, Invitrogen, Cat # 12371-019).  Real-time PCR was performed in triplicate using 60ng RNA equivalent of cDNA per sample, with SCA3 primers (5’- CAGGACAGAGTTCACATCCATGT-3’ and 5’-GCCTTACCTAGATCACTCCCAAGT-3’) and TaqMan probe sequence (5’-CCAGCCACCAGTTCAG-3’) and 18S human ribosomal RNA specific primers (Applied Biosystems, Cat # 4308329).  Expression levels were normalized using the 18s expression level in each sample, then relative levels of SCA3 expression determined.  Error bars represent technical variation of triplicates of each sample.

**Dpld homologues, protein family and phylogenetic tree**

The closest homologs of *dpld* were found by NCBI tBLASTn search. Human *lin-41* is the closest with 48% identity and 63% similarity in NHL domain. Then, worm lin-41 comes next with 41% identity and 57% similarity and rat lin-41 with 47% identity and 63% similarity. Dappled seems to closer to CG15105 with 39% identity and 56% similarity then Mei-P26 that has 31% identity and 47% similarity or Brat that 28% identity and 42% similarity over NHL repeats. Protein domain search for each conserved homolog was done using Pfam (<http://www.sanger.ac.uk/Software/Pfam/search.shtml>).

Since many of the RBCC-NHL family members have coiled coil domain, we used Macstripe version 2 (<http://www.york.ac.uk/depts/biol/units/coils/mstr2.html>) to predict coiled coil domain in Dpld and other family members. The Dpld phylogenetic tree was built using the NHL domain amino acid sequence in MacVector 7.0 (Genetics Computer Group, Madison WI USA), the numbers indicate the proportion of different amino acid sequence in the alignment (Neighbor joining method, distance is uncorrected and proportional gap distribution).

# Analysis of retinal degeneration:

# Flies bearing the disease gene presence or absence of *UAS-dpld* or the *EP* insertion allele *dpld^JM265^* were analyzed at 1 day and 21 day using corneal optical neutralization technique [1] as described [2]. At least 20 eyes per genotype were scored, 10 ommatidia per eye were counted. All experiments were repeated at least 3 times independently.

# References

S1. Franceschini N (1972) Pupil and pseudopupil in the compound eye of *Drosophila*. In: Wehner R, editor. Information Processing in the Visual Systems of arthropods. Berlin: Springer. pp. 75-82.

S2. Bilen J, Liu N, Burnett BG, Pittman RN, Bonini NM (2006) MicroRNA pathways modulate polyglutamine-induced neurodegeneration. Mol Cell 24: 157-163.

S3. Chan HY, Warrick JM, Gray-Board GL, Paulson HL, Bonini NM (2000) Mechanisms of chaperone suppression of polyglutamine disease: selectivity, synergy and modulation of protein solubility in Drosophila. Hum Mol Genet 9: 2811-2820.

S4. Lim J, Hao T, Shaw C, Patel AJ, Szabo G, et al. (2006) A protein-protein interaction network for human inherited ataxias and disorders of Purkinje cell degeneration.Cell 125: 801-814.

S5. Boeddrich A, Gaumer S, Haacke A, Tzvetkov N, Albrecht M, et al. (2006) An arginine/lysine-rich motif is crucial for VCP/p97-mediated modulation of ataxin-3 fibrillogenesis. EMBO J 25: 1547-1558.

S6. Frank DJ, Edgar BA, Roth MB (2002) The Drosophila melanogaster gene brain tumor negatively regulates cell growth and ribosomal RNA synthesis. Development 129: 399-407.

S7. Kazemi-Esfarjani P, Benzer S (2000) Genetic suppression of polyglutamine toxicity in Drosophila. Science 287: 1837-1840.

S8. Fernandez-Funez P, Nino-Rosales ML, de Gouyon B, She WC, Luchak JM, et al. (2000) Identification of genes that modify ataxin-1-induced neurodegeneration. Nature 408: 101-106.

S9. Rorth P, Szabo K, Texido G (2000) The level of C/EBP protein is critical for cell migration during Drosophila oogenesis and is tightly controlled by regulated degradation. Mol Cell 6: 23-30.

S10. Kraut R, Menon K, Zinn K (2001) A gain-of-function screen for genes controlling motor axon guidance and synaptogenesis in Drosophila. Curr Biol 11: 417-430.

S11. Dai P, Akimaru H, Ishii S (2003) A hedgehog-responsive region in the Drosophila wing disc is defined by debra-mediated ubiquitination and lysosomal degradation of Ci. Dev Cell 4: 917-928.
