# Supplementary material for: Genome-Wide Screen for Modifiers of Ataxin-3 Neurodegeneration in Drosophila
Source: PLoS Genet. 2007 Oct 19;3(10):e177. doi: 10.1371/journal.pgen.0030177 (PMC2041992; doi:10.1371/journal.pgen.0030177)
Supplement: Supplementary file 2 [file pgen.0030177.st001.doc]

Supplemental Table S1: Summary of genetic modifiers of Ataxin-3

| Table S1: Genetic Modifiers of Ataxin-3 | | | | | | | | |
| --- | --- | --- | --- | --- | --- | --- | --- | --- |
|  | **Gene** | **Allele(s)** | | | **Insertion site** | **Orientation and position** | | Confirmation of  over-expression |
| **CLASS 1: CHAPERONES** | | | | | | | | |
| 1 | Hsp68/ CG5436 | E407 | | | -426 bp | same, within 5'UTR | | Northern |
| 2 | DnaJ-1/ CG10578 | B345.2 | | | -632 bp | same, within predicted 5'UTR | | UAS-DnaJ-1 transgenes suppress [S3]; [S7] |
|  |  | EP411 | | | -600 bp | same, within predicted 5'UTR | | Insertion site, orientation and overexpression confirmation from [S8] |
| 3 | CG8448 | E1050 | | | -15,838 bp | same, within predicted 5'UTR of CG8848-RD | | Northern |
| 4 | CG14207 | EP1348 | | | -349 bp | same, upstream of 5'UTR | | Northern |
| 5 | Tpr2/ CG4599 | EB7-1A | | | -311 bp | same, upstream of 5'UTR | | UAS-dTPR2 suppresses [S7] |
| **CLASS 2: UBIQUITIN PATHWAY** | | | | | | | | |
| 6 | CG11700 | EP1384 | | | -560 bp | same, upstream of predicted 5'UTR | | RT-PCR |
| 7 | Ubp64E/ CG5486 | E213-1A | | | -4051 bp | same, upstream of 5'UTR; inserted within 5'UTR of DnaJ-1 | | EP3187 also suppresses; [S9]/ RT-PCR |
| 8 | CG8209 | B3-Sa | | | -96 bp | same, within 5'UTR | | Northern |
| 9 | Faf/ CG10372 | E659 | | | -339 bp | same, within predicted 5'UTR | | Northern |
| 10 | CG11033 | EP3093 | | | -1143 bp | same, upstream of 5'UTR | | Northern |
| **CLASS 3: MISCELLANEOUS FUNCTIONS** | | | | | | | | |
| 11 | emb/  CG13387 | E2-1A | | | -745 bp to emb | 2 adjacent hits or double-headed | | Western/RT-PCR |
|  |  | E128-1A | | | -1175 bp | same upstream of emb 5'UTR, within 3'UTR of fy | | RT-PCR |
| 12 | Sin3A/ CG8815 | B9-E | | | -4238 bp | same, in an intron | | RT-PCR |
| 13 | NFAT/CG11172 | EP1335 | | | +16,623 bp | within first intron | | Insertion site and upregulation from |
|  |  | EP1508 | | |  |  | | [S10] |
| 14 | dbr/  CG11371 | EP456 | | | -614 bp | same, upstream of 5'UTR | | Northern |
|  |  | EP9 | | | -459 bp | same, upstream of 5'UTR | | [S11] |
| 15 | Dpld/  CG1624 | JM120 | | | -3882 bp | same, upstream of 5'UTR | | Northern/RT-PCR |
|  |  | JM265 | | | -3769 bp |  | |  |
|  |  | E546 | | | -3782 bp |  | |  |
|  |  | EP2594 | | | -3769 bp |  | |  |
| 16 | orb2/CG5735 | B8-S | | | -3551 bp | same, in an intron | | RT-PCR |
| 17 | Imp/ CG1691 | EP1433 | | | -1548 bp | same, in an intron | | Insertion site and upregulation from [S10] |
| 18 | CG5009 | | B227.2 | -140 bp | | | same, upstream of 5'UTR | Northern |
