# Supplementary material for: Genome-Wide Screen for Modifiers of Ataxin-3 Neurodegeneration in Drosophila
Source: PLoS Genet. 2007 Oct 19;3(10):e177. doi: 10.1371/journal.pgen.0030177 (PMC2041992; doi:10.1371/journal.pgen.0030177)
Supplement: Supplementary file 3 [file pgen.0030177.st002.doc]

**Supplemental Table S2:** Loss of function deficiency regions and alleles of modifiers of Ataxin-3 neurodegeneration that were tested.

| **Table S2: Loss of function deficiency regions and alleles of modifiers of Ataxin-3 neurodegeneration that were tested.** | | | |
| --- | --- | --- | --- |
|  | **Gene** | **Deficiencies & alleles tested** | **Effect on SCA3trQ78 toxicity** |
| 1 | Hsp68/ CG5436 | Df(3R)Exel6197 | none |
| 2 | DnaJ-1/ CG10578 | Df(3L)CH20 | enhances |
|  |  | Df(3L)CH18 | enhances |
| 3 | CG8448 | Df(2R)Exel6063 | none |
|  |  | Df(2R)Jp5 | none |
|  |  | Df(2R)Jp7 | none |
| 4 | CG14207 | No alleles | ND |
| 5 | Tpr2/ CG4599 | Df(2L)r10 | enhances |
|  |  | Tpr2^KG08262 *^ | none |
| 6 | CG11700 | No alleles | ND |
| 7 | Ubp64E/ CG5486 | Df(3L)CH20 | enhances |
|  |  | Df(3L)CH18 | enhances |
| 8 | CG8209 | Df(3L)pbl-X1 | none |
|  |  | Df(3L)ZP1 | none |
| 9 | Faf/ CG10372 | Df(2L)Exel7071 | none |
|  |  | Df(2L)OD15 | none |
| 10 | CG11033 | Df(3R)Exel6258 | none |
|  |  | Df(3R)p-XT9 | none |
|  |  | CG11033^KG04325*^ | none |
| 11 | emb/CG13387 | emb^3 *^ | enhances |
|  |  | Df(2L)TE29Aa-16 | enhances |
|  |  | emb^k16715 *^ | none |
| 12 | Sin3A/ CG8815 | Sin3A^08269*^ | none |
| 13 | CG11172 | Df(1)HA92 | none |
| 14 | dbr/CG11371 | Df(2L)PM44 | enhances |
| 15 | dpld/CG1624 | dpld^l(2)k14202*^ | none |
|  |  | dpld^l(2)k08815*^ | none |
| 16 | orb2/CG5735 | Df(3L)Exel6113 | none |
|  |  | orb2^BG02373*^ | none |
| 17 | Imp/ CG1691 | Df(1)HC133 | none |
|  |  | Df(1)v-L11 | none |
|  |  | Df(1)HA92 | none |
| 18 | CG5009 | Df(2R)Exel7150 | none |
|  |  | Df(2R)14H10W-35 | none |
| * Extent to which alleles may decrease gene function relative to a deficiency line was not determined. | | | |
|  |  |  |  |
