# Supplementary material for: Genome-Wide Screen for Modifiers of Ataxin-3 Neurodegeneration in Drosophila
Source: PLoS Genet. 2007 Oct 19;3(10):e177. doi: 10.1371/journal.pgen.0030177 (PMC2041992; doi:10.1371/journal.pgen.0030177)
Supplement: Supplementary file 4 [file pgen.0030177.st003.doc]

Supplemental Table S3: Protein Interactions of Ataxin-3 Genetic Modifiers

| **Human gene / Gene ID (*Drosophila* gene)** | **Degree of interaction to Ataxin-3** | Notes |
| --- | --- | --- |
| **HSPA1A/3303 (orthologue of fly CG5436/Hsp68)** | (4°) | heat shock 70kDa protein 1A |
| HSPA1A is a direct interaction protein of Ataxin-1. | | |
| **DNAJB4/ 11080 (orthologue of CG10578/DnaJ-1)** |  | DnaJ (Hsp40) homolog, subfamily B, member 4 |
| not found in 2-hybrid screen; other DNAJ proteins were found | | |
| **Other DNAJ proteins:** |  |  |
| **DNAJA3/9093** | (3°) | DnaJ (Hsp40) homolog, subfamily A, member 3 |
| DNAJA3 is a direct interaction partner of Ataxin-1 | | |
| **DNAJB1/3337** | (5°) | DnaJ (Hsp40) homolog, subfamily B, member 1 |
| **DNAJB11/51726** | (5°) | DnaJ (Hsp40) homolog, subfamily B, member 11 |
| **DNAJC3/5611** | (7°) | DnaJ (Hsp40) homolog subfamily C member 3 |
| **CRYAB/1410 (orthologue of fly CG14207)** | (4°) | crystallin, alpha B |
| **DNAJC7/7266 (orthologue of fly CG4599/Tpr2)** |  | DnaJ (Hsp40) homolog, subfamily C, member 7 |
| not found in yeast two hybrid screen | | |
| **Other Tpr domain containing proteins:** |  |  |
| **PEX5/5830** | (5°) | peroxisomal biogenesis factor 5 |
| **ST13/6767** | (5°) | Hsc70-interacting protein (Hip) |
| **DNAJC3/5611** | (7°) | DnaJ (Hsp40) homolog subfamily C member 3 |
| **STUB1/10273** | (5°) | Carboxy terminus of Hsp70-interacting protein |
| **FLJ21908/79657** | (2°) | hypothetical protein |
| **UBC/7316 (orthologue of fly CR11700/poly-ubiquitin)** | (4°) | ubiquitin C |
| **USP47/55031 (orthologue of fly Ubp64E/CG5486)** |  | Ubiquitin-specific-processing protease 47 |
| No direct interaction with USP47 | | |
| **Other USP proteins:** |  |  |
| **USP7/7874** | (2°) | Ubiquitin-specific-processing protease 7 |
| USP7 is a direct interaction partner of Ataxin-1. | | |
| **USP2/9099** | (6°) | Ubiquitin-specific-processing protease 2 |
| **USP4/7375** | (5°) | Ubiquitin-specific-processing protease 4 |
| **USP5/8078** | (5°) | Ubiquitin-specific-processing protease 5 |
| **USP6/9098** | (3°) | Ubiquitin-specific-processing protease 6 |
| **USP6NL/ 9712** | (4°) | Ubiquitin-specific-processing protease 6NL |
| **USP8/9101** | (4°) | Ubiquitin-specific-processing protease 8 |
| **USP52/9924** | (2°) | Ubiquitin-specific-processing protease 52 |
| **USP53/54532** | (3°) | Ubiquitin-specific-processing protease 53 |
| **USP54/159195** | (3°) | Ubiquitin-specific-processing protease 54 |
| USP54 is a direct interaction partner of Atrophin-1 |  |  |
| **UBXD2/23190 (Orthologue of fly CG8209)** | (2°) | Stress activated protein kinase substrate 1 |
| UBA/UBX domain | | |
| (interacts with VPC; VCP is a direct Ataxin-3 interactor protein) | | |
| **UBXD8/23197 (Orthologue of fly CG10372/Faf)** |  | Homo sapiens UBX domain containing 8 |
| No direct interaction found | | |
| **FBXL11/22992 (Orthologue of fly CG11033)** |  | Homo sapiens F-box protein 11 |
| No direct interaction found | | |
| **Other F box proteins:** |  |  |
| **FBXW5/54461** | (3°) | F-box and WD-40 domain protein 5 |
| **FBXW7/55294** | (4°) | F-box and WD-40 domain protein 7 |
| **FBXL18/80028** | (3°) | F-box and leucine-rich repeat protein 18 |
| **FBXO18/84893** | (5°) | F-box and helicase helicase protein 18 |
| **FBXO28/23219** | (3°) | F-box protein 28 |
| **FBXL20/84961** | (2°) | F-box and leucine-rich repeat protein 20 |
| **FBXO42/54455** | (4°) | F-box only protein 42 |
| **Xpo1/7514 (Orthologue of fly CG13387/emb)** | (4°) | nuclear export protein |
| Xpo1 in the same complex as Nup proteins | | |
| **Nup proteins found in 2-hybrid screen:** |  |  |
| **NUP88/4927** | (2°) | 88kDa nuclear pore complex protein |
| **NUP214/8021** | (3°) | 214kDA nuclear pore complex protein |
| **SIN3A/25942 (Orthologue of fly Sin3A)** | (3°) | Histone deacetylase complex subunit Sin3a |
| SIN3A is a direct interaction partner of SMN1, mutated in Spinal Muscular Atrophy | | |
| **NFAT5/10725 (Orthologue of fly NFAT)** |  | Nuclear factor of activated T-cells 5 |
| No NFAT5 interaction | | |
| **Other NFAT proteins found in 2-hybrid screen:** |  |  |
| **NFATC2/4773** | (4°) | Nuclear factor of activated T-cells, cytoplasmic 2 |
| **NFATC4/4776** | (4°) | Nuclear factor of activated T-cells, cytoplasmic 4 |
|  |  |  |
| **TRIM71/131405 (Orthologue of fly CG1624/dpld)** |  | abnormal cell LINeage LIN-41 |
| NO Lin41 interaction found | | |
| **Other TRIM proteins that share domains with Dpld:** |  |  |
| **TRIM32/22954** | (3°) | Tripartite motif containing protein 32 |
| B box, coiled coil and NHL domain -TRIM32 is a direct interaction partner of Ataxin-1 | | |
| **TRIM37/4591** | (2°) | tripartite motif-containing 37 |
| B box and coled coil domain, peroxisomal protein |  |  |
|  |  |  |
| **CREB2/1386 (Orthologue of fly CG5735/orb2)** |  | Cyclic AMP response element-binding protein 2 |
| No interaction with CREB2 | | |
| **Other CREB-related proteins:** |  |  |
| **CREBBP/1387** | (2°) | CREB binding protein |
| **CREB1/1385** | (3°) | cAMP response element-binding protein |
| **CREB3L3/84699** | (3°) | CAMP responsive element binding protein 3-like 3 |
|  |  |  |
| **IGF2BP2/10644 (Orthologue of fly Imp)** |  | insulin-like growth factor 2 mRNA binding protein 2 |
| No interaction with IGF2BP | | |
| **Other KH domain proteins:** |  |  |
| **KHDRBS2/202559** | (2°) | KH domain-containing, RNA-binding, signal transduction-associated protein 2 |
| **KHDRBS3/10659** | (4°) | KH domain containing, RNA binding, signal transduction associated 3 |
| **QKI/9444** | (5°) | quaking homolog, KH domain RNA binding |
| **ACOX1/51 (Orthologue of fly CG5009)** | (4°) | peroxisomal acyl-coenzyme A oxidase |
|  |  |  |
